# Antibacterial effect of crude extract and metabolites of *Phytolacca americana* on pathogens responsible for periodontal inflammatory diseases and dental caries

**DOI:** 10.1186/1472-6882-14-343

**Published:** 2014-09-20

**Authors:** Jayanta Kumar Patra, Eun Sil Kim, Kyounghee Oh, Hyeon-Jeong Kim, Yangseon Kim, Kwang-Hyun Baek

**Affiliations:** School of Biotechnology, Yeungnam University, Gyeongsan, Gyeongbuk, 712-749 Republic of Korea; Wildlife Genetic Resources Center, National Institute of Biological Resources, Incheon, 404-708 Republic of Korea; Ensoltek Co., Ltd, Techno 10-ro 51, Yuseong-gu, Daejeon, 305-510 Republic of Korea

**Keywords:** Kaempferol, *Phytolacca americana*, *Porphyromonas gingivalis*, Quercetin, *Streptococcus mutans*

## Abstract

**Background:**

The oral cavity is the store house of different species of microorganisms that are continuously engaged in causing diseases in the mouth. The present study was conducted to evaluate the antibacterial potential of crude extracts of the aerial parts of *Phytolacca americana* and its natural compounds against two oral pathogens, *Porphyromonas gingivalis* and *Streptococcus mutans*, which are primarily responsible for periodontal inflammatory diseases and dental caries, as well as a nonpathogenic *Escherichia coli*.

**Methods:**

Crude extract and fractions from the aerial parts of *P. americana* (0.008–1.8 mg/mL) were evaluated for their potential antibacterial activity against two oral disease causing microorganisms by micro-assays. The standard natural compounds present in *P. americana*, kaempferol, quercetin, quercetin 3-glucoside, isoqueritrin and ferulic acid, were also tested for their antibacterial activity against the pathogens at 1–8 μg/mL.

**Results:**

The crude extract was highly active against *P. gingivalis* (100% growth inhibition) and moderately active against *S. mutans* (44% growth inhibition) at 1.8 mg/mL. The chloroform and hexane fraction controlled the growth of *P. gingivalis* with 91% and 92% growth inhibition at a concentration of 0.2 mg/mL, respectively. Kaempferol exerted antibacterial activity against both the pathogens, whereas quercetin showed potent growth inhibition activity against only *S. mutans* in a concentration dependent manner.

**Conclusion:**

The crude extract, chloroform fraction, and hexane fraction of *P. americana* possesses active natural compounds that can inhibit the growth of oral disease causing bacteria. Thus, these extracts have the potential for use in the preparation of toothpaste and other drugs related to various oral diseases.

## Background

The mouth cavity contains many microorganisms responsible for various infections and inflammatory diseases [1]. The most common types of pathological diseases in the mouth are periodontal diseases and dental caries [2]. These diseases are caused by various plaque forming bacteria such as *Streptococcus mutans*, *Porphyromonas gingivalis*, *Prevotella intermedia, Actinobacillus* sp., and *Fusobacterium* sp. [2–4], which reside in the oral cavity. Periodontal disease is a common inflammatory disease inside the oral cavity caused by a complex biofilm of periodontopathic and resident commensal bacteria species that form an integral part of the disease along with the environment and host related factors [5, 6]. Periodontal diseases are primarily caused by a group of anaerobic Gram-negative bacteria including *P. gingivalis, P. intermedia, Actinobacillus* and *Fusobacterium* sp. [4]. The formation of dental caries is caused by the accumulation and colonization of oral microorganisms, especially *S. mutans* [3], which adhere to and colonize the surface of teeth, as well as to other oral bacteria that cause dental plaque disease, such as *Actinomyces* sp.*, Actinobacillus* sp. and *Prevotella* sp. [2, 3].

Dental treatments in many countries are expensive and not easily accessible; therefore, people have turned to the use of medicinal plants in the form of composition of tooth paste, or simply chewing the plants directly to protect the teeth from diseases [7, 8]. Different plant species of medicinal importance have successfully been included in mouthwashes and toothpastes in many developing countries [1, 9, 10]. Moreover, public research programs and dental care companies are screening medicinal plants and their extracts for their ability to control the pathogens *P. gingivalis* and *S. mutans*, which are the root cause of most oral cavity diseases.

*Phytolacca americana* L., which is commonly known as tropical pokeweed, belongs to the family Phytolaccaceae. *P. americana* L. is an herbaceous perennial plant distributed throughout most of North and South America, Africa and Asia [11, 12]. Because of their antimicrobial, anti-inflammatory, anticancer and stimulatory effects, herbal preparations of this plant have been used extensively in traditional medicines in South and Central America for the treatment of many disorders including glandular swelling with heat and inflammation, syphilitic bone pain, sore throat, quinsy and diphtheria [11, 13, 14]. The use of this plant in herbal medicine is typically based on its folklore and traditional evidence. Scientific evidence on the medicinal potential and some types and nature of bioactive compounds present in this plant has been reported [12].

In the process of performing research on screening of various medicinal effects of invasive plants in the Republic of Korea funded by the National Institute of Biological Resources (NIBR, http://www.nibr.go.kr), the overwhelming antibacterial potential of the extracts of *P. americana* against the oral pathogens *P. gingivalis* and *S. mutans* were evaluated*.* In this study, therefore, various experiments investigating a range of concentrations of crude extract from *P. americana* and the individual fractions were conducted to evaluate the potential for development of new natural antibacterial medicines without affecting the normal microflora of the mouth cavity by testing the antibacterial effect of the extract on nonpathogenic *Escherichia coli*.

## Methods

### Preparation of crude extract of *P. americana*

The aerial parts (leaves and soft stem) of the wild herb *P. americana* were collected from the local area of Inchean (Sindori, Wongjin-gun, Inchon, Korea) during June 2013. Accurate identification of *P. americana* was conducted by an experienced taxonomist Jeong-Eun Han, and specimens were stored at the Natural Products Bank, Wildlife Genetic Resources Center at the NIBR (voucher number NIBRVP0000413856). The plant parts were washed twice with tap water and then dried in an oven for 3 days at 40°C. Following drying, samples were chopped into small pieces (about 2.5 cm) using a straw cutter, after which 500 g of dried pieces were extracted in 80% methanol using an ultrasonic apparatus. The solvent was removed under vacuum, and 17.24% is the yield of the extract. This methanolic extract was further suspended in H_2_O, then partitioned successively with *n*-hexane, chloroform, ethanol, and *n*-butanol [15]. All crude extract and fractions were passed through a nylon membrane filter (0.2 μm pore size), freeze-dried, dissolved in 5% dimethyl sulfoxide (DMSO) and stored at -20°C freezer until further use. All chemicals used in this experiment were of analytical grade and purchased from Sigma-Aldrich (St. Louis, USA).

### Screening of antibacterial activity of *P. americana*

Two oral pathogenic bacteria, *P. gingivalis* W83 (ATCC BAA-1703™) and *S. mutans* UA159 (ATCC 700610™), and one nonpathogenic bacterium, *E. coli* DH5α, used in the study were procured from the American Type Culture Collection (ATCC). *P. gingivalis* and *S. mutans* were cultured anaerobically in brain-heart infusion broth media supplemented with hemin and menadione and tryptic soy broth media, respectively [16, 17], and *E. coli* DH5α strain was cultured in Luria-Bertuni (LB) broth media.

The antibacterial activity was evaluated by standard micro-assays using conventional sterile polystyrene microplates [18]. The assay mixture contained 50 μL of inocula and 50 μL of the tested extract/fraction/compounds at different concentrations (0.2–1.8/0.008–0.2 mg/mL and 1–8 μg/mL) respectively. Additionally, 50 μL of media with 50 μL of inoculum was used as a control and 50 μL of media with 50 μL of 5% DMSO was used as solvent control. All experiments were repeated three times and all concentrations of the extract and compounds were measured in triplicate. The natural compounds included kaempferol, quercetin, quercetin 3-glucoside, isoquercitrin and ferulic acid. The microplates were incubated at 37°C for 24 h, and the growth of bacteria was determined by measuring the optical density (OD) at 630 nm using an ELISA microplate reader (FilterMax F5 multi-mode microplate reader, Molecular Devices, Sunnyvale, CA, USA). Minimum inhibitory concentration (MIC) of the extract was taken as the lower concentration of the extract showing no visible growth of the organism. Finally, the percentage of bacterial growth inhibition (GI) was calculated using the following formula:


Where C_Abs_ is the absorbance of the control treatment and T_Abs_ is the absorbance of samples treated with different extracts.

### Statistical analysis

All experiments were conducted three times and the results were expressed as the means of three samples ± the standard deviation (SD). Analysis of the variance was determined by Duncan's test using the Statistical Analysis Software (SAS) version 9.2 (SAS Inc., Cary, USA).

## Results

The bactericidal effects of the crude extract and different fractions of the extracts of *P. americana* were evaluated against two oral pathogenic bacteria, *P. gingivalis* and *S. mutans*, and one nonpathogenic bacteria, *E. coli* DH5α (Table 1). At the highest concentration used (1.8 mg/mL), the crude extract was very active against *P. gingivalis* (100% growth inhibition) and moderately active against *S. mutans* (44% growth inhibition) (Table 1). However, the crude extract inhibited only 6% of the growth of *E. coli* DH5α at the same concentration (Table 1). After further decrease in the concentration of the crude extract by three and five times (0.6 or 0.2 mg/mL, respectively), significant suppression of the growth of *P. gingivalis* by 100 or 88%, respectively, was still observed. The crude extract showed low antibacterial activity against *S. mutans* and *E. coli* DH5α at the same concentrations (Table 1).

**Table 1:** **Antibacterial activity of crude extract and fractions of** ***Phytolacca americana*** **against the tested bacteria**

Plant fractions	Concentration	***P. gingivalis***	***S. mutans***	***E. coli***DH5α
		Growth inhibition (%)		
Crude extract	0.2 mg/mL	88 ± 19.06^b*#^	13 ± 21.66^yz^	2 ± 3.94
	0.6 mg/mL	100 ± 0.39^a^	35 ± 25.01^xy^	3 ± 3.20
	1.8 mg/mL	100 ± 0.41^a^	44 ± 35.29^x^	6 ± 4.45
	MIC	0.2 mg/mL	1.8 mg/mL	-
Hexane Fr.	0.008 mg/mL	0 ± 0.00^e^	2 ± 16.46^z^	0 ± 0.00
	0.04 mg/mL	26 ± 2.36^d^	0 ± 0.00^z^	0 ± 0.00
	0.2 mg/mL	92 ± 0.56^ab^	62 ± 1.49^x^	0 ± 0.00
	MIC	0.2 mg/mL	0.2 mg/mL	-
CHCl_3_ Fr.	0.008 mg/mL	0 ± 0.00^e^	0 ± 0.00^z^	0 ± 0.00
	0.04 mg/mL	0 ± 0.00^e^	0 ± 0.00^z^	0 ± 0.00
	0.2 mg/mL	91 ± 2.00^ab^	56 ± 10.52^x^	0 ± 0.00
	MIC	0.2 mg/mL	0.2 mg/mL	-
EtOAc Fr.	0.008 mg/mL	0 ± 0.00^e^	0 ± 0.00^z^	7 ± 1.90
	0.04 mg/mL	0 ± 0.00^e^	1 ± 0.00^z^	2 ± 1.38
	0.2 mg/mL	46 ± 5.63^c^	0 ± 0.00^z^	0 ± 0.00
BuOH Fr.	0.008 mg/mL	0 ± 0.00^e^	0 ± 0.00^z^	10 ± 0.83
	0.04 mg/mL	0 ± 0.00^e^	0 ± 0.00^z^	6 ± 1.05
	0.2 mg/mL	4 ± 1.55^e^	0 ± 0.00^z^	0 ± 0.00

^*^Data are expressed as the mean ± SD.

^#^Values in the same column with different superscript letters are significantly different (*p* < 0.001).

After measuring the activity of crude extract from *P. americana* against *P. gingivalis*, the crude extract was further fractioned using four different solvents. When the fractions were tested at 0.2 mg/mL, the hexane and chloroform fraction significantly suppressed the growth of *P. gingivalis* by 92% and 91%, respectively. When the concentration was further diluted by five times (0.04 mg/mL), the antibacterial effects of the hexane and chloroform fractions against *P. gingivalis* decreased significantly to 26% and 0%, respectively. Evaluation of the extract of *P. americana* revealed that the hexane and chloroform fractions inhibited the growth of *S. mutans* by 62% and 56%, respectively, at 0.2 mg/mL. However, the fractions did not exert an antibiotic effect on *E. coli* DH5α (Table 1).

**Figure 1:**
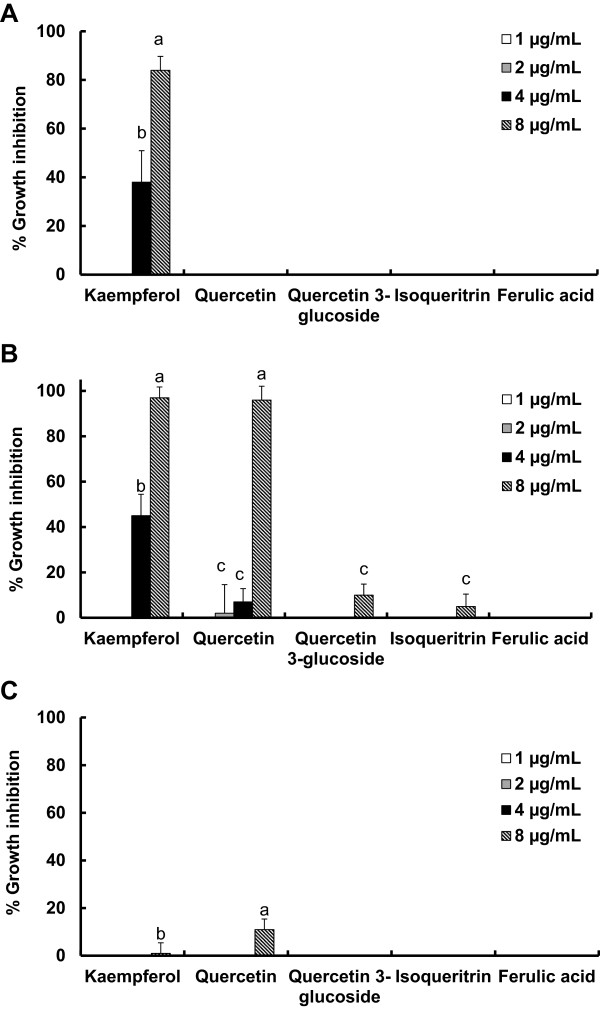
**Antibacterial activity of the natural compounds present in** ***Phytolacca americana*** **against the tested bacteria. (A)** *Porphyromonas gingivalis*; **(B)** *Streptococcus mutans*; **(C)** *Escherichia coli* DH5α strain. Different letters on the bars are significantly different at *p* < 0.001.

The antibacterial effects of some selected natural compounds in *P. americana* L. such as kaempferol, quercetin, quercetin 3-glucoside, isoquercitrin and ferulic acid were also tested at different concentrations (1–8 μg/mL) against the two oral pathogenic and one non-pathogenic bacteria. Only kaempferol displayed higher antibacterial effects against both *P. gingivalis* and *S. mutans* and low activity against *E. coli* (Figure 1). At 8 μg/mL, kaempferol exhibited 84% antibacterial activity against *P. gingivalis*, which was reduced to 38% when the concentration of the compound was, diluted two times (Figure 1). Further dilution resulted in loss of activity of the compound against the pathogen. Similarly, kaempferol showed 97% and 45% growth inhibition against *S. mutans* at 8 μg/mL and 4 μg/mL, respectively (Figure 1). Quercetin exhibited 96% growth inhibition against *S. mutans* at 8 μg/mL, but no effect was observed against *P. gingivalis* at any of the tested concentrations.

Quercetin 3-glucoside and isoqueritrin showed very little growth inhibition activity against *S. mutans* and no activity against the other tested organisms (Figure 1). None of the natural compounds of the plant exerted significant inhibition activity against the nonpathogenic *E. coli*.

## Discussion

Human pathogenic microorganisms have developed resistance to drugs owing to the extensive use of commercial synthetic antibacterial drugs in large quantity without proper medical prescriptions and tests. This condition has raised alarm in most developed and developing countries and the scientists are forced to search an alternative to these compounds, often in the form of natural medicines from resources such as plants. It is well known that *in vitro* evaluation of plant species with ethno-medicinal potential is the first step towards development of a new eco-friendly and effective drug against any type of infectious disease. In the present study, a similar attempt was made to search for potential bioactive compounds from *P. americana* for treatment of various periodontal inflammatory diseases and dental caries that are caused by pathogenic anaerobic bacteria (*P. gingivalis* and *S. mutans*). Crude extract of *P. americana* inhibited the growth of *P. gingivalis* by 100% at 1.8 and 0.6 mg/mL (Table 1). Even diluted concentrations of 0.2 mg/mL inhibited growth by 88%. Additionally, crude extract exerted moderate effects of 44% and 35% on *S. mutans* at 1.8 and 0.6 mg/mL (Table 1), while it had very low activity against non-pathogenic *E. coli.*

With an aim to identify the active fraction in which most of the antibacterial compounds are separated, the crude methanol extract was fractioned using hexane, chloroform, ethanol and butanol and then tested against the three aforementioned bacteria. Among all the fractions, hexane and chloroform fractions showed greater inhibition of the growth of *P. gingivalis* and *S. mutans* (Table 1). Significant antibacterial activity of the fraction against *P. gingivalis* might have been due to the fact that the natural bioactive compounds responsible for the antibacterial activity are mostly extracted in both the solvents and these active compounds may be able to penetrate the thick cell walls through general diffusion channels formed by the bacterial porins present therein [2, 7, 19, 20], and affect the bacterial enzymes like gingipain that are responsible for survival and virulence of *P. gingivalis* [21, 22] resulting in cellular lysis. However these extracts do not affect the non-pathogenic bacteria which may be due to hindrance of penetration through the outer cell wall and absence of specific enzyme in the bacteria. This mode of action of the plant extract against the specific bacteria may be due to its secondary mode of action against the bacterial enzymes instead of acting on the cell wall of the bacteria [21, 22].

Five most important natural compounds present in *P. americana* with possible antibacterial properties were selected and evaluated for their antibacterial activity against oral pathogens. Kaempferol showed significant antibacterial activity against both the oral pathogens in a concentration dependent manner (Figure 1), whereas only quercetin showed potent growth inhibition activity against *S. mutans.* This might have been due to easy penetration of the quercetin molecule into the Gram positive bacterial cells of *S. mutans*, resulting in disruption of the cell wall and cytoplasmic membrane [23]. Compounds such as kaempferol have previously been reported to exhibit inhibitory activity against oral pathogens [24, 25]. Cai and Wu [26] demonstrated the potent growth-inhibitory activity of kaempferol against the periodontal pathogens *P. gingivalis* and *P. intermedia*. Liberio et al. [27] also reported that the active compound present in geopropolis produced by the stingless bee, *Melipona fasciculate* Smith and that possess antibacterial activity against oral pathogens is Quercetin. Similar effects of quercetin on oral infectious pathogens were also reported by Shu et al. [28]. The crude extract and its fractions exerted potent activity against bacterial pathogens relative to the pure natural compounds, suggesting synergistic properties of compounds that display promising antibiotic results in mixture and lose its activity when applied individually.

The antibacterial effects of the plant extract justified its ethnomedicinal uses in traditional medicine [11, 14, 29, 30]. Similar investigations of the use of plants for the treatment of oral cavity infections have also been reported [2, 31]. Additionally, the plant extract and its fractions were not very effective at inhibiting the nonpathogenic bacteria, *E. coli* DH5α, which indicates that, use of the plant extract as medicine or in toothpaste will only affect oral pathogenic bacteria, and not the normal flora of the mouth cavity.

## Conclusions

Herbal plant extracts are a rich source of natural chemical compounds that have been utilized in various applications on the basis of their biological activities. Active compounds present in plants have shown positive results against oral pathogenic microbes and can thus facilitate management of various inflammatory diseases related to the mouth and dental caries. The present study is a preliminary attempt to investigate the antibacterial properties of aerial parts of *P. americana* against *P. gingivalis* and *S. mutans*. Based on the results of the present study, the plant fractions can be used in herbal toothpaste and medicine for the treatment of periodontal disease and dental caries. However, further research is needed to identify and characterize the active molecules responsible for their antibacterial properties and to determine its potential for use in pharmaceutical industries.
